# Mr. Walker's Table of Comparative Quantities in Pharmaceutical Compositions

**Published:** 1817-03

**Authors:** 


*For the London Medical and Physical Journal.*

Mr. WALKER's *Table of Comparative Quantities in Pharmaceutical Compositions.*

SHOULD the following table, which I have myself found extremely useful as an occasional reference, be deemed not unworthy your notice, you will have the goodness to insert sert it. There is, I apprehend, more of practical utility in it, than ingenuity in its construction.

*A Table for ascertaining, in any given or assumed Quantity, in any Pharmaceutical Composition, the proportional Parts of the various Articles which enter into such Composition.*

| 1 | 2 | 3 | 4 | 5 | 6 | 7 | 8 | 9 |
|---|---|---|---|---|---|---|---|---|
| | ℥ ʒ gr. | ℥ ʒ gr. | ℥ ʒ gr. | ℥ ʒ gr. | ℥ ʒ gr. | ʒ gr. | ʒ gr. | ʒ gr. |
| ℥ 32 | 16 | 8 | 4 | 2 | 1 | 4 | 2 | 1 |
| 16 | 8 | 4 | 2 | 1 | 4 | 2 | 1 | 30 |
| 15 | 7 4 | 3 6 | 1 7 | 7 30 | 3 45 | 1 53 | 56 | 28 |
| 14 | 7 | 3 4 | 1 6 | 7 | 3 30 | 1 45 | 53 | 26 |
| 13 | 6 4 | 3 2 | 1 5 | 6 30 | 3 15 | 1 38 | 48 | 24 |
| 12 | 6 | 3 | 1 4 | 6 | 3 | 1 30 | 45 | 22 |
| 11 | 5 4 | 2 6 | 1 3 | 5 30 | 2 45 | 1 22 | 41 | 20 |
| 10 | 5 | 2 4 | 1 2 | 5 | 2 30 | 1 15 | 37 | 19 |
| 9 | 4 4 | 2 2 | 1 1 | 4 30 | 2 15 | 1 7 | 34 | 17 |
| 8 | 4 | 3 | 1 | 4 | 2 | 1 | 30 | 15 |
| 7 | 3 4 | 1 6 | 7 | 3 30 | 1 45 | 53 | 26 | 13 |
| 6 | 3 | 1 4 | 6 | 3 | 1 30 | 45 | 23 | 11½ |
| 5 | 2 4 | 1 2 | 5 | 2 30 | 1 15 | 37 | 19 | 9½ |
| 4 | 2 | 1 | 4 | 2 | 1 | 30 | 15 | 7½ |
| 3 | 1 4 | 6 | 3 | 1 30 | 45 | 22 | 11 | 5½ |
| 2 | 1 | 4 | 2 | 1 | 30 | 15 | 7½ | 3¾ |
| 1 | 4 | 2 | 1 | 30 | 15 | 8 | 4 | 2 |
| ʒ 7 | 3 30 | 1 45 | 52 | 26 | 13 | 6½ | 3¼ | 1½ |
| 6 | 3 | 1 30 | 45 | 22 | 11 | 5½ | 2¾ | 1¼ |
| 5 | 2 30 | 1 15 | 37 | 19 | 9½ | 4¾ | 2¼ | 1⅛ |
| 4 | 2 | 1 | 30 | 15 | 7½ | 3¾ | 1⅞ | 1 |
| 3 | 1 30 | 45 | 22 | 11 | 5½ | 2¾ | 1¼ | ½ |
| 2 | 1 | 30 | 15 | 7½ | 3¾ | 1¾ | ¾ | ⅜ |
| 1 | 30 | 15 | 7½ | 3¾ | 1¾ | ¾ | ⅜ | ¼ |
| gr. 50 | 25 | 12½ | 6¼ | 3⅛ | 1½ | ¾ | ⅜ | ¼ |
| 40 | 20 | 10 | 5 | 2½ | 1¼ | ½ | ¼ | ⅛ |
| 30 | 15 | 7½ | 3¾ | 1⅞ | 1 | ½ | ¼ | ⅛ |
| 20 | 10 | 5 | 2½ | 1¼ | ½ | ¼ | ⅛ | 1/16 |
| 10 | 5 | 2½ | 1¼ | ½ | ¼ | ⅛ | 1/16 | 1/32 |
| 9 | 4½ | 2¼ | 1⅛ | ½ | ¼ | ⅛ | 1/16 | 1/32 |
| 8 | 4 | 2 | 1 | ½ | ¼ | ⅛ | 1/16 | 1/32 |
| 7 | 3½ | 1¾ | ⅞ | ½ | ¼ | ⅛ | 1/16 | 1/32 |
| 6 | 3 | 1½ | ¾ | ⅜ | ¼ | ⅛ | 1/16 | 1/32 |
| 5 | 2½ | 1¼ | ½ | ¼ | ⅛ | 1/16 | 1/32 | 1/64 |
| 4 | 2 | 1 | ½ | ¼ | ⅛ | 1/16 | 1/32 | 1/64 |
| 3 | 1½ | ¾ | ⅓ | ⅙ | 1/12 | 1/24 | 1/48 | 1/96 |
| 2 | 1 | ½ | ¼ | ⅛ | 1/16 | 1/32 | 1/64 | |
| 1 | ½ | ¼ | ⅛ | 1/16 | 1/32 | 1/64 | | |

N. B.—In the application of this table, consisting of nine perpendicular columns, it must be constantly recollected, that a pint of any liquid in measure is sixteen ounces, but that a pound in weight, of either solid or liquid, is twelve ounces; the ounce in each is divided alike; the minim measure, in liquids, corresponding to the grain in solids; and that two drops, ordinarily speaking, of any spirituous tincture, are equal but to one minim.

The

The use of this table may be exemplified thus:—Suppose it be required to know the strength of a drachm of tincture of jalap, in which eight ounces of jalap have been infused in thirty-two ounces (two liquid pounds or pints) of spirit. First, find out the larger quantity of the medicine given, which, in this instance, is the first quantity in column 1, viz. thirty-two ounces; secondly, find out the quantity, the strength of which is to be ascertained, on the same horizontal line, viz. one drachm, which is in column 9; thirdly, find out in column 1, the quantity of the article infused in the larger quantity of the liquid, viz. eight ounces, carry the eye on the same line to the quantity given in the column of 1 drachm, viz. 9, which is fifteen grains, exhibiting the quantity of jalap infused, or the strength of one drachm of tincture of jalap, according to the first given formula. The application of the table is after the same manner in the composition of solid as well as liquid medicines, as powders, pills, &c.; for, showing the proportions of each ingredient to the other, in any given or assumed quantity. Where unequal quantities occur, that is, such as do not appear in the perpendicular column of fig. 1, as twenty-four ounces for instance, two columns must be gone through, viz. 16 ounces and 8 ounces, after a similar manner; or one half, or any other proportion of the primary quantities, may be assumed, and the process conducted after the same manner.

In this manner, the proportional parts of each, or the principal ingredient only, in any given composition, whether solid or liquid, may at any time be readily found, after a little previous practice.

In the construction of this table, mathematical exactness has been dispensed with in some minute instances, such not being conveniently practicable, and unnecessary in the application of it.

The application of this table will be found extremely easy, and almost instantaneous, after a little practice, and will, I have no doubt, be found useful to others, as it has been extremely so to myself.

I have adopted the ***minim measure*** in this table, and elsewhere, conformably to the directions given in the London Pharmacopœia of 1809; but for my opinion respecting its practical utility, and other circumstances connected with the subject, see some observations of mine "On Weights and Measures," Medical Journal for July 1814, page 16,—observing, at page 17, for "five drops," to read "five minims."

---

P.S.—More and "more last words" still, from Mr. Woodham, since my last communication, I perceive. When this gentleman fastens upon any one, methinks he is not easily choaked off; —thorough game, at least, it must be allowed.

I noticed with much satisfaction your remarks on the communications from your Nestorean Correspondent, so *grossly* reprobated by Mr. Woodham in the 210th Number of your Journal, for which I beg you will receive my unfeigned acknowledgments.

I am

I am too conscious of having monopolized to myself more than my professional share in your valuable Journal; but, whilst I am countenanced by your approbation, to which I can add that of others, which I have noticed in print, I may, in future, venture to attribute Mr. W.'s *churlish remarks* to an immature or distempered judgment, and apply, in my own mind, the following couplet to him:—

> " All seems infected, that th' infected spy,
> " As all looks yellow to the jaundiced eye."

I shall, therefore, leaving him to breathe out his spleen in unheeded soliloquies, pursue occasionally, (but with more reserve,) with your indulgence, my professional career, merely leaving this observation by the way, viz. that Mr. W. (poor deluded gentlemen!) seems entirely to have mistaken the design of my communications, and, as I apprehend, his own qualification, which is, that he should *profit* by them, not *comment* upon them.

There is one error, and one only, respecting my communications, which Mr. W. has fallen into, that, possibly, no one but myself could correct, viz. that in furnishing my " lengthy" papers, he concludes I must have leisure, were I so disposed, to have paid more attention to *his* criticisms. The fact is, that I sacrifice the time, which I can very ill spare, to furnish my communications, presuming, rightly I hope, that I am performing a useful duty, but it by no means follows, that I should make a still greater sacrifice of my time and mind, in answering his petulance.

